# Supplementary material for: RESTORE: Robust intEnSiTy nORmalization mEthod for multiplexed imaging
Source: Commun Biol. 2020 Mar 9;3:111. doi: 10.1038/s42003-020-0828-1 (PMC7062831; doi:10.1038/s42003-020-0828-1)
Supplement: Supplementary file 1 — Supplementary Information [file 42003_2020_828_MOESM1_ESM.pdf]

Supplementary Information

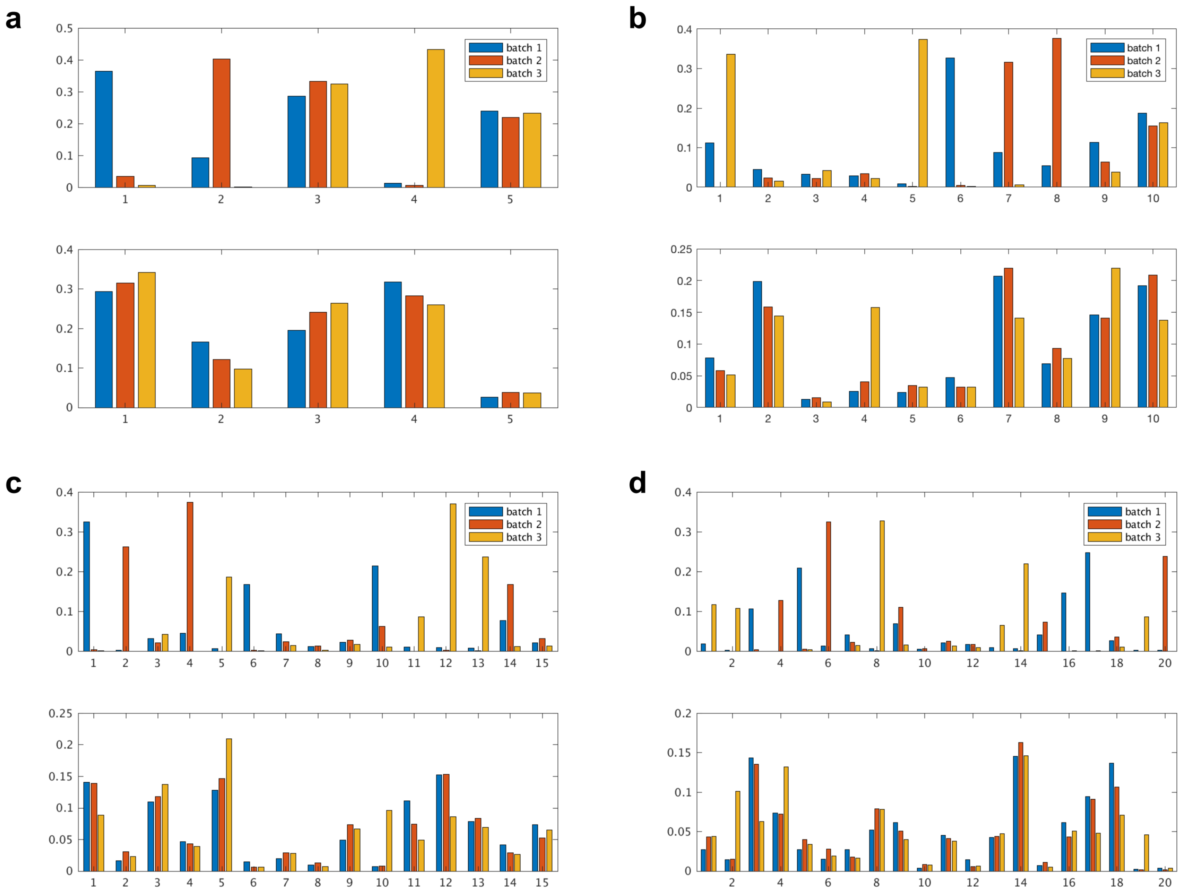

Supplementary Figure 1: Example of cell composition comparison based on unsupervised clustering result between without normalization and with normalization with varying the number of cluster (**a** ($N = 5$), **b**($N = 10$), **c**($N = 15$) and **d**($N = 20$)). For each figure, top row shows the result without normalization and bottom row shows the result with normalization where x-axis represents clustered group ID and y-axis represents their cell population. With normalization approaches, each cluster group shows uniform distribution from each batch.

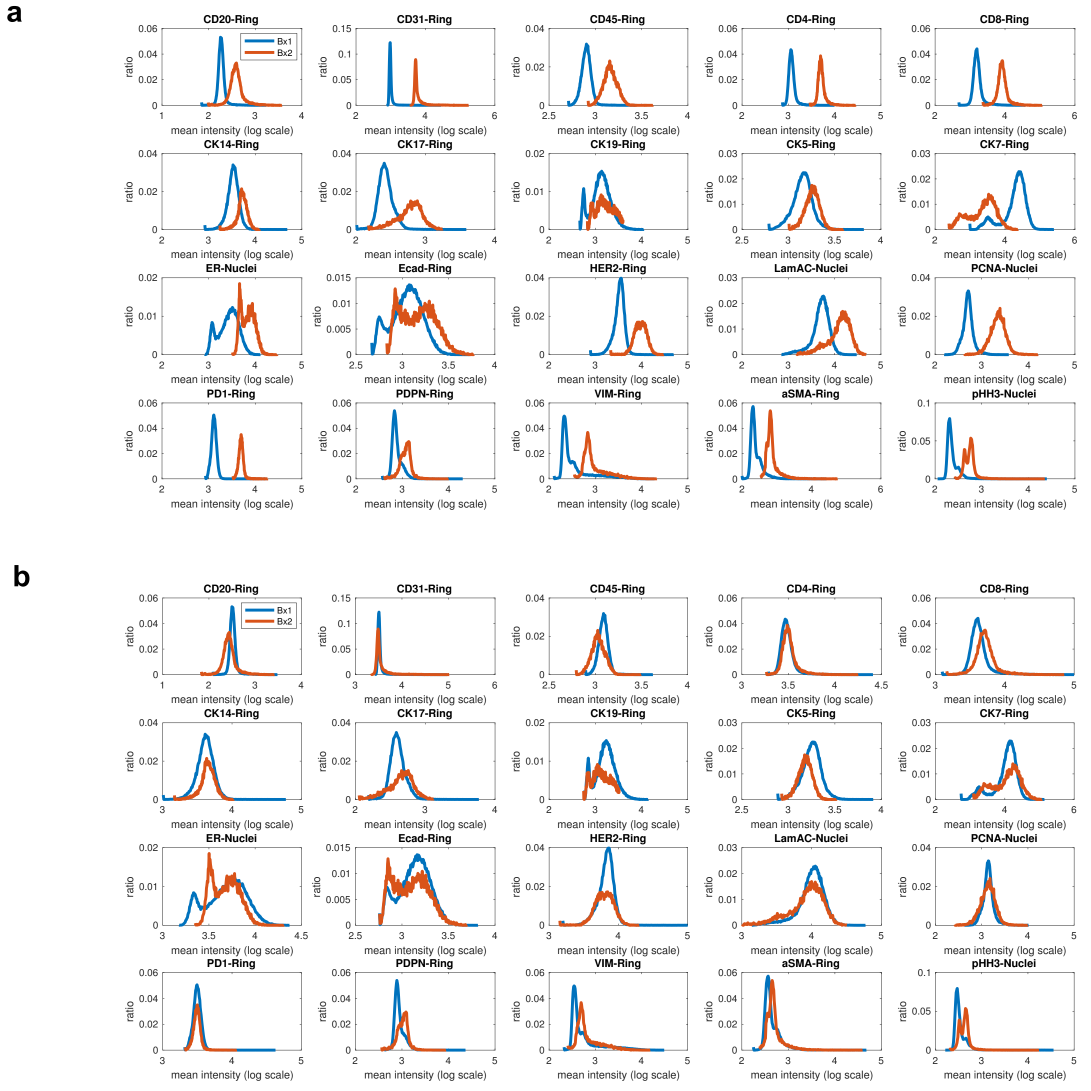

Supplementary Figure 2: CycIF Intensity distribution across Bx 1 (**a**) and Bx 2 (**b**) where top figure shows before normalization and bottom figure shows after normalization based on inferred background signal.

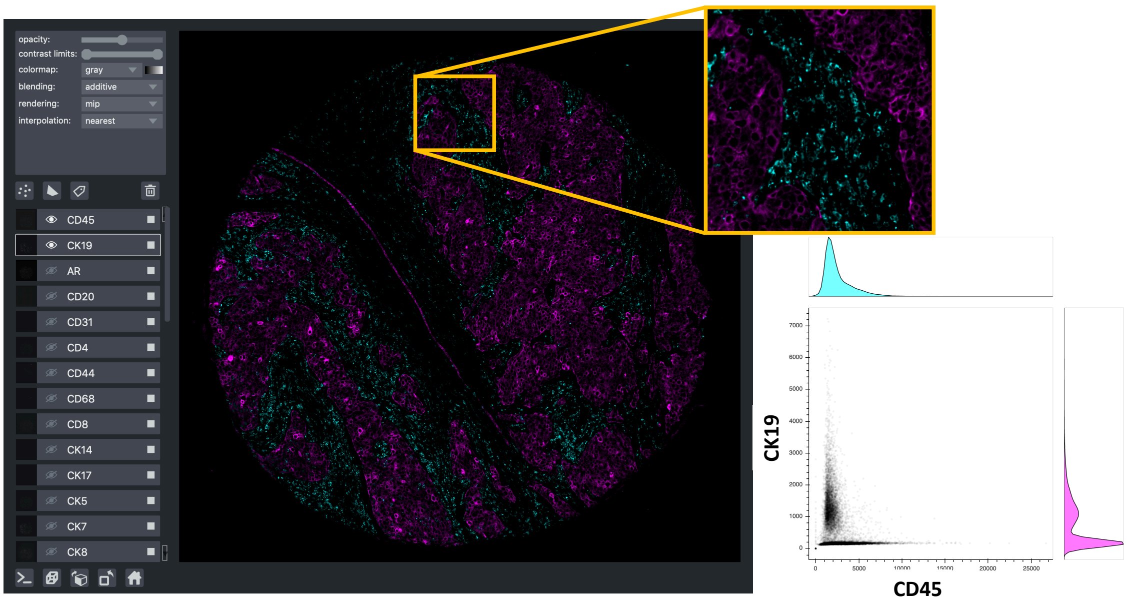

Supplementary Figure 3: A simple multi-dimensional image viewer based on Napari [26] will be useful to validate mutually exclusive biomarker pairs. Here, we visualize immune marker (CD45) and cancer marker (CK19) with scatter plot which shows clear mutual exclusiveness.

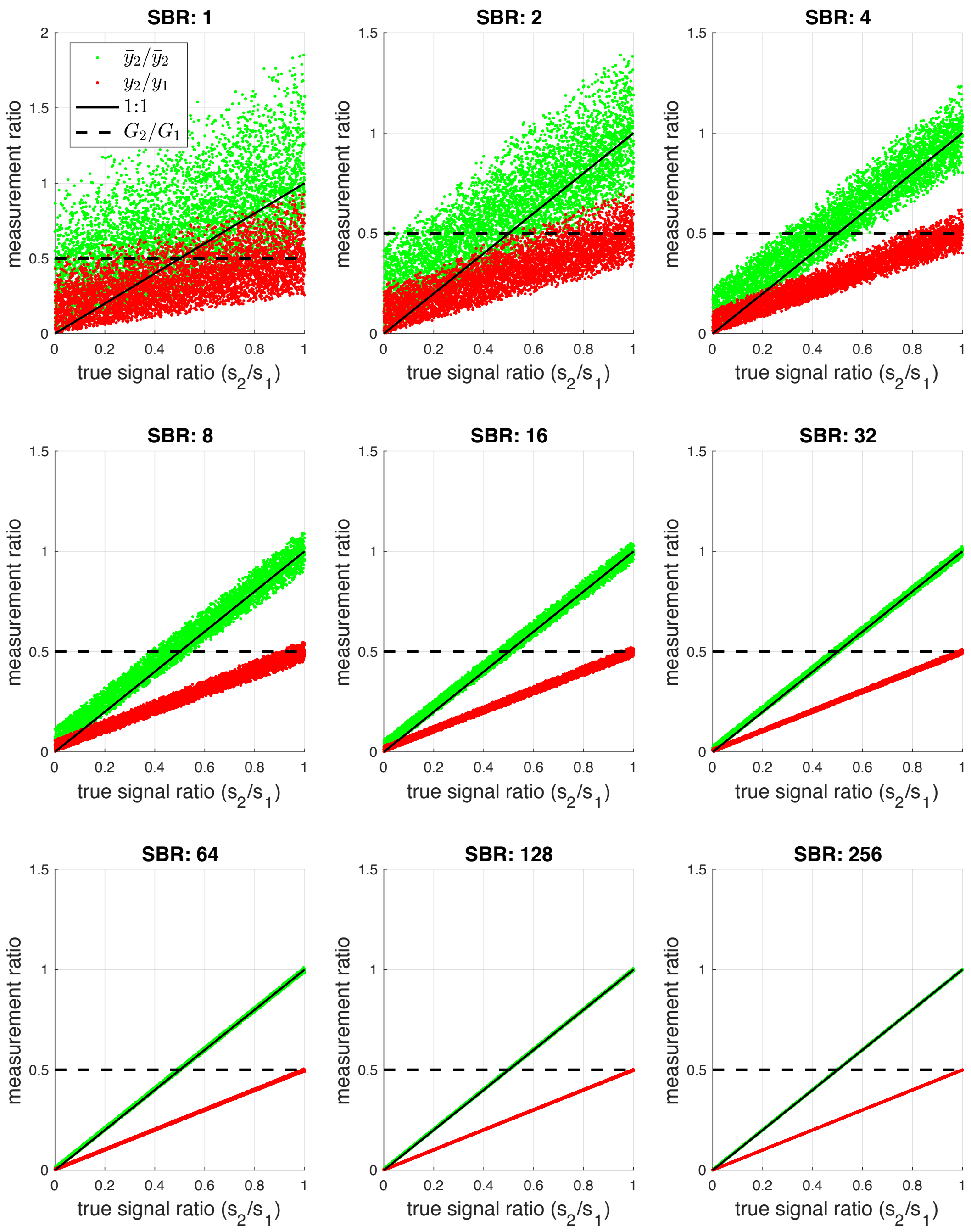

Supplementary Figure 4: Comparison between true signal ratio and measurement signal ratio with and without normalization by changing signal to background ratio (SBR): as SBR increases, the normalized signal ratio is close to the true signal ratio. On the other hand, without normalization, measurement signal ratio is proportional to gain G_2/G_1 (0.5, in this simulation setting).

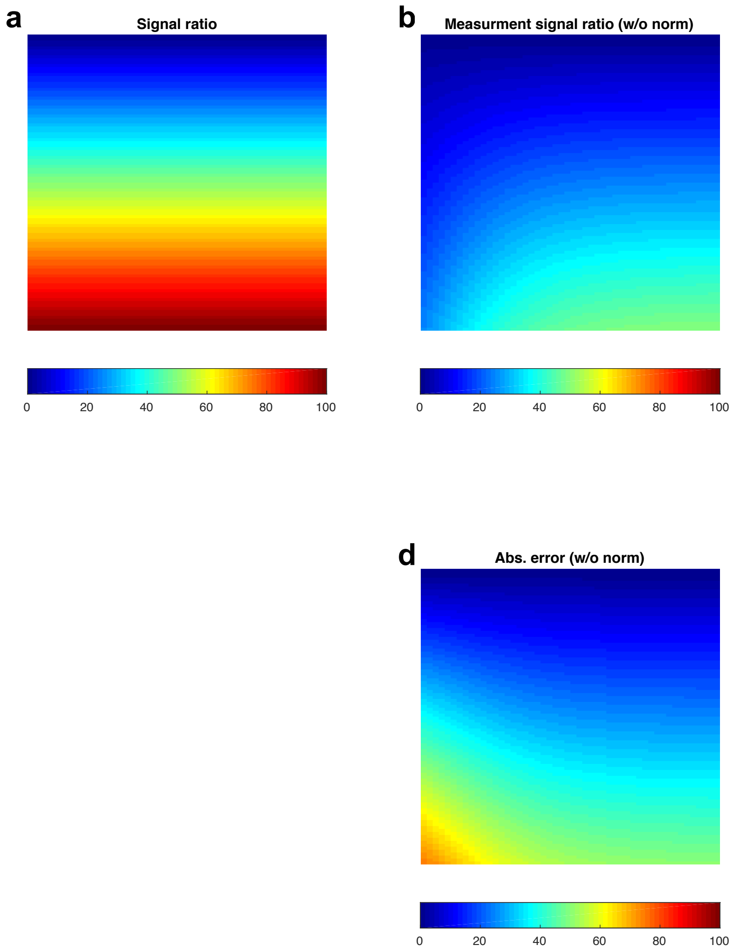

Supplementary Figure 5: A heat map representation: (**a**) true signal ratio, (**b**) measurement signal ratio [w/o norm], (**c**) measure signal ratio [w/ norm], (**d**) error [w/o norm], (**e**) error [w/ norm] where color represents signal ratio (low in blue and high in red). From left to right across columns, signal to background ratio (SBR) increases and thus, measurement signal ratio with normalization is close to the true signal ratio.

	**Data-driven approach**					**Knowledge-based approach**				
Markers	**1st**	**2nd**	**3rd**	**4th**	**5th**	**1st**	**2nd**	**3rd**	**4th**	**5th**
AR	CD31	HER2	CK7	CD45	LamB2	-	-	-	-	-
aSMA	CK19	HER2	CD68	PgR	H3K27	CK19	CK7	CD68		
CD20	CD31	HER2	CK7	CD68	PgR	CK19	CK7	CK14	CK5	
CD3	CK7	HER2	CD31	CK19	CD68	CK19	CK7	CK14	CK5	
CD4	HER2	CK19	CK7	CD31	H3K27	CK19	CK7	CK14	CK5	
CD44	CK19	CD68	Ecad	PgR	HER2	-	-	-	-	-
CD45	CK7	HER2	CD31	CK19	LamB2	CK19	CK7	CK14	CK5	
CD68	CK19	PgR	CK14	Ecad	H3K27	CK19	CK7	CD31		
CD8	CK7	HER2	CD31	CK19	LamB2	CK19	CK7	CK14	CK5	
CK14	CD68	Ki67	ColI	cPARP	GrNZB	CD31	CD68	Vim		
CK17	CD68	CK14	PgR	CK19	CD44	CD31	CD68	Vim		
CK19	CD68	CD44	Vim	CD31	CD4	CD68	CD4	CD31		
CK5	CD68	PgR	CK19	CD44	Ecad	CD31	CD68	Vim		
CK7	CD31	Vim	CD4	CD45	CD3	CD68	CD4	CD31		
CK8	CD31	CD68	Vim	CD4	HER2	CD68	CD4	CD31		
ColI	CK14	CD68	CK19	PgR	CK5	CK19	CK7	CK14	CK5	
ColIV	CD68	PgR	CK19	HER2	CD31	CK19	CK7	CK14	CK5	CD68
cPARP	CK14	CD68	GRNZB	PgR	Ki67	-	-	-	-	-
Ecad	CD68	CD44	CK14	Vim	PgR	CD68	CD4	CD31		
ER	CD31	HER2	CK7	CD68	Vim	CD68	CD4	CD31		
FoxP3	CD68	HER2	CK19	PgR	CD31	CK19	CK7	CK5		
GRNZB	CK14	Ki67	cPARP	ColI	CD68	CK19	CK7	CK5	CD31	
H3K27	CD68	HER2	CD44	CD31	PgR	-	-	-	-	-
H3K4	CD31	CD68	HER2	CK19	PgR	-	-	-	-	-
LamB2	HER2	CD31	CK7	CK19	CD68	-	-	-	-	-
LaminAC	CD68	PgR	CK19	CD44	CK14	-	-	-	-	-
PCNA	CK7	CD45	CD31	HER2	CD3	-	-	-	-	-
PD1	CD68	HER2	CD31	PgR	CK19	CK19	CK7	CK14	CK5	CD31
PDPNP	CD68	CK19	HER2	PgR	CK14	CK19	CK7	CK14	CK5	CD68
pERK	CD68	HER2	CD31	PgR	CK14	-	-	-	-	-
PgR	CD68	CK14	HER2	CD44	CK19	CD68	CD4	CD31		
pHH3	GRNZB	CK14	CD68	ColI	Ki67	-	-	-	-	-
pRB	CK14	CD68	PgR	CK19	GRNZB	-	-	-	-	-
pS6	CD68	CK14	PgR	CK19	CD44	-	-	-	-	-
Vim	HER2	CK19	CK7	Ecad	H3K27	CK19	CK7	CD68		
HER2	CD31	Vim	CD4	PgR	CD44	CD68	CD4	CD31		
Ki67	CK14	CD68	GRNZB	CD44	PgR	-	-	-	-	-

Supplementary Table 1: A list of mutually exclusive marker pairs based on (left panel) data-driven and (right panel) biologically-known information. Red color on the left panel indicates that matched pairs exist on the right panel. Green color on the right panel indicates that matched pairs exist on the left panel.
